# Oxytocin as a Modulator of Synaptic Plasticity: Implications for Neurodevelopmental Disorders

**DOI:** 10.3389/fnsyn.2018.00017

**Published:** 2018-06-19

**Authors:** Keerthi Thirtamara Rajamani, Shlomo Wagner, Valery Grinevich, Hala Harony-Nicolas

**Affiliations:** ^1^The Department of Psychiatry, Icahn School of Medicine at Mount Sinai, New York City, NY, United States; ^2^The Seaver Autism Center for Research and Treatment, Icahn School of Medicine at Mount Sinai, New York City, NY, United States; ^3^Sagol Department of Neurobiology, Faculty of Natural Sciences, University of Haifa, Haifa, Israel; ^4^Schaller Research Group on Neuropeptides at German Cancer Research Center (DKFZ), Central Institute of Mental Health and Cell Networks Cluster of Excellence, University of Heidelberg, Heidelberg, Germany

**Keywords:** oxytocin, synaptic plasticity, neurodevelopmental disorder, autism spectrum disorder (ASD), animal models for ASD

## Abstract

The neuropeptide oxytocin (OXT) is a crucial mediator of parturition and milk ejection and a major modulator of various social behaviors, including social recognition, aggression and parenting. In the past decade, there has been significant excitement around the possible use of OXT to treat behavioral deficits in neurodevelopmental disorders, including autism spectrum disorder (ASD). Yet, despite the fast move to clinical trials with OXT, little attention has been paid to the possibility that the OXT system in the brain is perturbed in these disorders and to what extent such perturbations may contribute to social behavior deficits. Large-scale whole-exome sequencing studies in subjects with ASD, along with biochemical and electrophysiological studies in animal models of the disorder, indicate several risk genes that play an essential role in brain synapses, suggesting that deficits in synaptic activity and plasticity underlie the pathophysiology in a considerable portion of these cases. OXT has been repeatedly shown, both *in vitro* and *in vivo*, to modify synaptic properties and plasticity and to modulate neural activity in circuits that regulate social behavior. Together, these findings led us to hypothesize that failure of the OXT system during early development, as a direct or indirect consequence of genetic mutations, may impact social behavior by altering synaptic activity and plasticity. In this article, we review the evidence that support our hypothesis.

## Introduction

Behaviors are driven by diverse sets of functionally and anatomically connected brain regions that form brain circuits (Insel and Fernald, 2004; Goodson and Kabelik, 2009; Averbeck and Costa, 2017; Kohl et al., 2017; Roseberry and Kreitzer, 2017; Yang and Wang, 2017). Communications within brain circuits are not hard-wired but rather constantly adapting to the environment via neuromodulatory mechanisms. These mechanisms involve various neuromodulators, including neuropeptides, which exert their effect on neural ensembles to construct and modulate the circuit function and to shape a specific behavior (Marder, 2012; Nusbaum and Blitz, 2012). The last two decades have been enriched with studies exploring the behavioral effects of the pro-social oxytocin (OXT) neuropeptide (Heinrichs et al., 2003, 2009; Kirsch et al., 2005; Guastella et al., 2008a,b; Marsh et al., 2010; Guastella and MacLeod, 2012) and its mechanisms of action (Blume et al., 2008; Jurek et al., 2012; van den Burg et al., 2015). In parallel, several clinical and preclinical studies have focused on the therapeutic potential of OXT, mainly to treat social behavior deficits (Guastella and Hickie, 2016; Wagner and Harony-Nicolas, 2017). However, little attention has been paid to the possible implication of the OXT system in neurodevelopmental disorders and to whether perturbation in OXT may contribute to the social behavior phenotype. The objective of this review is to provide a framework for the role OXT plays in modulating synaptic plasticity and its implication in neurodevelopmental disorders. We begin by summarizing studies that examined the role of OXT in regulating synaptic plasticity underlying behavior. We then highlight studies that report specific alterations in the OXT system in rodent models of neurodevelopmental disorders. Finally, we explore the potential convergence between the OXT system and genes associated with neurodevelopmental disorders, focusing on the *SHANK3* gene.

### The Oxytocin System

OXT is a neuropeptide that is exclusively synthesized in neurons residing in the paraventricular (PVN), supraoptic (SON) and accessory nuclei (AN) of the hypothalamus. These nuclei harbor two major types of OXT-producing cells: magno- and parvocellular neurons (Althammer and Grinevich, 2017). Magnocellular OXT neurons project to the posterior pituitary for OXT release into systemic circulation and concomitantly send axonal collaterals to a large proportion of forebrain regions. Parvocellular (or preautonomic) OXT neurons differ in their projections to the midbrain, brainstem and spinal cord, thus controlling autonomic and metabolic processes as well as processing of nociceptive and non-nociceptive information (see reviews: (Althammer and Grinevich, 2017; Boll et al., 2017; Poisbeau et al., 2017)).

In rodents, OXT action is conveyed through the OXT receptor (OXTR), whose expression shows a significant overlap with axon terminals of OXT neurons within the brain (Grinevich et al., 2016; Marlin and Froemke, 2017). OXTR is a G-protein-coupled receptor that is capable of enhancing (Knobloch et al., 2012; Stoop, 2012) or reducing (Eliava et al., 2016) the excitability of neural cells via distinct mechanisms, including its dual coupling to Gq or Go/Gi proteins (Gravati et al., 2010; Busnelli et al., 2012). Among the intracellular signaling pathways activated by the OXTRs is the mitogen-activated protein kinase (MAPK) cascade (van den Burg and Neumann, 2011), whose role in regulating specific behaviors is now beginning to emerge. For example, it has been demonstrated that the OXT anxiolytic effect requires OXTR/MEK/ERK signaling (Blume et al., 2008; Jurek et al., 2012; van den Burg et al., 2015), and that this anxiolytic pathway strictly requires the influx of extra-cellular calcium through transient receptor potential vanilloid (TRPV) channels (van den Burg et al., 2015).

### Oxytocin, Synaptic Plasticity and Behavior

Long-term potentiation (LTP) and long-term depression (LTD) are the most common forms of long-term synaptic plasticity. Both are long-lasting changes in synaptic strength induced by certain patterns of synaptic activity (Cooke and Bliss, 2006). LTP and LTD are considered as putative synaptic mechanisms that mediate learning and memory (Redondo and Morris, 2011). The effect of OXT on LTP was first demonstrated by Dubrovsky et al. (2002) in the rat hippocampus. The authors examined the effect of intracerebroventricular (ICV) administration of OXT (1 μg) on LTP induction *in vivo* using high-frequency tetanic stimulation in the dentate gyrus (DG) of anesthetized rats. They found that, in the presence of OXT, tetanic stimulation induced LTD rather than the expected LTP. We have reported a similar effect of OXT in the medial nucleus of the amygdala (MeA) in anesthetized rat. Specifically, we examined the effect of ICV OXT administration (1 μg) on synaptic plasticity induction by tetanic stimulation of the accessory olfactory bulb (AOB) (Gur et al., 2014) and demonstrated that OXT strongly augments LTD induction in the AOB-MeA pathway. The MeA is an essential component of the brain network that subserves social recognition memory (SRM) (Ferguson et al., 2002), a subtype of social memory that enables subjects to remember and distinguish individual conspecifics (Gheusi et al., 1994). In agreement with previous findings (Ferguson et al., 2001; Lukas et al., 2013), our study validated that OXT in the MeA is crucial for SRM and further suggested that the OXT mediated LTD in the AOB-MeA pathway is involved in consolidating long-term SRM (Gur et al., 2014). We have recently demonstrated that ICV administration of CRF-related peptide urocortin3 or 17β-estradiol 45 min before OXT administration induced LTP rather than LTD in the MeA in response to AOB stimulation, a result that suggests a bidirectional long-term plasticity in the AOB-MeA synaptic pathway (Frankiensztajn et al., 2018).

In 2003, Tomizawa et al. (2003) reported that OXT perfusion (1 μM) of mouse hippocampal slices enhanced the ability of subthreshold synaptic stimulation to induce long-lasting LTP (L-LTP) at Schaffer collateral-CA1 synapses. The authors also demonstrated that this induction was mediated by the activation of the MAPK cascade and phosphorylation of cyclic AMP-responsive element binding protein (CREB), suggesting that OXT induced neuronal plasticity in the hippocampus is transcription-dependent. In an attempt to correlate these findings with behavior, the authors demonstrated that ICV administration of OXT in virgin mice improved long-term spatial learning, a result that aligned with a previous discovery showing that spatial memory is enhanced during pregnancy, delivery and lactation, situations when OXT levels are substantially high (Kinsley et al., 1999). Similarly, Lin et al. (2012) demonstrated that endogenous OXT contributes to the maintenance of late but not early phase LTP, which was induced by subthreshold stimulation. Furthermore, they showed that the OXT-induced enhancement of LTP is OXTR dependent and involves an EGFR-mediated rapid and persistent increase in the local translation of an atypical protein kinase C (PKC) isoform, thus describing a mechanism for OXT-dependent LTP. Notably, stress is known to have a prolonged negative effect on memory and synaptic plasticity (Kim et al., 2015). In rats, an uncontrollable stress experience following unpredictable and unescapable shocks causes impairment in hippocampal-dependent memory tasks and leads to deficits in both LTP and LTD (Foy et al., 1987; Shors et al., 1989; Xu et al., 1997; Kim et al., 2001). The effect of OXT on stress-induced impairments in synaptic plasticity and cognition has been recently addressed in two separate studies by the same group (Lee et al., 2015; Park et al., 2017). These studies demonstrated that administration of intranasal OXT before or after the stress event could reverse the LTP and LTD deficits observed in hippocampal slices as well as improve spatial memory impairments by activating OXTRs and regulating ERK activity.

The nucleus accumbens (NAc) is a key component of the mesocorticolimbic dopamine reward circuit and is known to be a target for synaptic plasticity-associated changes induced by drugs of abuse (Luscher and Malenka, 2011). Dolen et al. (2013) have recently shown that within the NAc, a region that is also implicated in social reward, OXT acts as a social reinforcement signal, and blocking OXTRs in the NAc inhibits the establishment of a preference for social cues. Furthermore, they showed that bath application of OXT (1 μM) induced a presynaptic LTD in NAc medium spiny neurons caused by decreased presynaptic neurotransmitter release probability. Using viral genetic tools, they demonstrated that presynaptic OXTRs on serotonergic axon terminals, arriving from the dorsal raphe nucleus to the NAc, are required for social reward and OXT-induced LTD, which they found to be dependent on the coordinated activity of OXT and serotonin (Dolen et al., 2013).

The effect of OXT on synaptic plasticity has also been studied in mouse brain slices from other brain regions. In mouse slices of infralimbic medial prefrontal cortex, OXT (100 nM) produced a significant suppression of basal glutamatergic neurotransmission through reduction of presynaptic glutamate release and lead to conversion of the activity dependent LTD to LTP. This OXT-dependent conversion is NMDA receptor-dependent and requires synaptic insertion of calcium-permeable AMPA receptors (Ninan, 2011). In slices of the AOB, OXT (0.2–2 μM) facilitated the induction of a NMDA receptor-dependent LTP in reciprocal synapses of excitatory mitral cells on inhibitory interneurons, thus enhancing inhibition on the mitral cells (Fang et al., 2008). In slices of the left auditory cortex, OXT induced LTP and led to increased spike firing (Mitre et al., 2016). Finally, although out of the scope of the current review that focuses on OXT and synaptic plasticity in the context of behavior, it is important to note that the effect of OXT on synaptic plasticity has been also studied in the context of pain, where stimulation of OXT-PVN neurons or intrathecal OXT administration was shown to reduce or prevent LTP in spinal dorsal horn neurons and transiently interrupt the long-lasting LTP-mediated mechanical hyperalgesia (DeLaTorre et al., 2009).

### Implications for Neurodevelopmental Disorders

**Figure 1:**
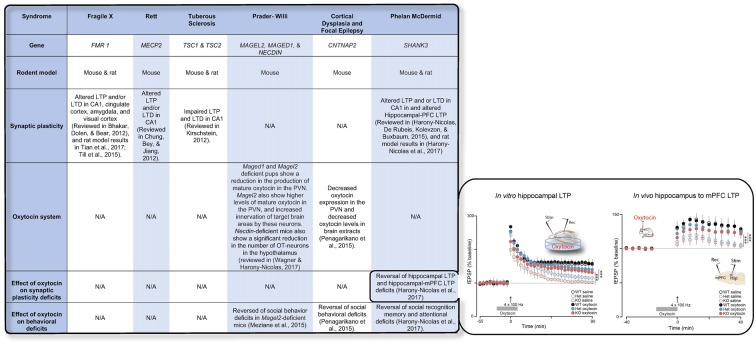
The table summarizes the available knowledge on synaptic plasticity deficits, alteration in the oxytocin (OXT) system, and the effect of OXT administration on behavioral and/or synaptic plasticity deficits in six syndromes associated with autism spectrum disorder (ASD). Inset shows the effect of OXT on synaptic plasticity deficits in the *Shank3*-deficient rat model. Reproduced from Figure 6, Harony-Nicolas et al., 2017; eLife, published under the Creative Commons Attribution 4.0 International Public License CC BY 4.0; (https://creativecommons.org/licenses/by/4.0/). *FMR1*, Fragile X mental retardation; *MECP2*, Methyl-CpG-binding protein 2; *TSC1/2*, Tuberous Sclerosis 1/2; *MAGEL2*, MAGE Family Member L2; *MAGED1*, MAGE Family Member D1; *CNTNAP2*, contactin associated protein like 2; *SHANK3*, SH3 And Multiple Ankyrin Repeat Domains 3; LTP, Long-term potentiation; LTD, Long-term depression; mPFC, medial prefrontal cortex; PVN, paraventricular nucleus.

Recent advances in genetic studies of autism spectrum disorder (ASD) and other neurodevelopmental disorder have implicated several risk genes that play an essential role in brain synapses (Xu et al., 2012; De Rubeis et al., 2014; Sanders et al., 2015), a finding that suggests that deficits in synaptic activity and plasticity may underlie the pathophysiology of these disorders in a considerable portion of the cases. Electrophysiological studies in animal models for ASD have supported this theory and have repeatedly shown that LTP and/or LTD are impaired in the vast majority of these models (Bhakar et al., 2012; Chung et al., 2012; Kirschstein, 2012; Harony-Nicolas et al., 2015, 2017; Till et al., 2015; Tian et al., 2017) (examples for are presented in Figure 1). The role that OXT plays in modulating synaptic plasticity led us to hypothesize that failure of the OXT system during early development may impact social behavior by altering synaptic plasticity in brain regions implicated in social behavior. Here we summarize several studies that propose that dysfunction in the OXT system early in life could account for the development of some of the social behavior symptoms.

### Prader-Willi Syndrome

Prader-Willi Syndrome (PWS) is a rare multisystem neurodevelopmental disorder that presents with abnormal clinical features during development, starting with severe hypotonia and feeding difficulties in infants followed by unrelenting feelings of hunger and consequently excessive eating and obesity problems later in life (Angulo et al., 2015). Individuals with PWS also present with intellectual disability and some features of ASD (Bennett et al., 2015). PWS results from the lack of expression of the paternal allele of several contiguous genes including *MKRN3*, *MAGEL2*, *MAGED1*, *NECDIN* and *SNURF-SNRPRN*. Notably, subjects with PWS have a significantly decreased number of PVN-OXT neurons and decreased levels of circulating OXT (Swaab et al., 1995; Hæybye, 2004). These alterations are suggested to underlie the obesity phenotype in PWS patients (reviewed in Sabatier et al., 2013). Mouse models for PWS present with several phenotypes, some of which mimic those observed in subjects with PWS. *Maged1*-deficient mice develop progressive obesity, show impaired social interaction and social memory and display alerted sexual behavior, increased anxiety and self-grooming. Notably, in these mice, the synthesis of mature OXT in the brain is also significantly decreased, and restoring OXT levels via acute peripheral administration of OXT reverses the social memory deficits (Dombret et al., 2012). Similarly, the *Magel2*-deficient pups also show a significant reduction in the levels of mature OXT peptide in the brain. These abnormalities are accompanied by deficits in social and learning behaviors that are reversed following subcutaneous administration of OXT at an early postnatal stage (Schaller et al., 2010; Meziane et al., 2015). Notably, *Magel2*-deficient mice also exhibit feeding difficulties, a phenotype that mirrors those observed in patients with PWS. Finally, in the *Necdin*-deficient mouse model, the number of hypothalamic OXT-neurons is also significantly decreased (Muscatelli et al., 2000). Together, these studies suggest that the alterations in OXT system may underlie the social behavior deficits observed in subjects with PWS and that OXT treatment may be beneficial to treat these deficits. Similarly, a recent clinical study showed that intranasal application of OXT in subjects with PWS under age of 6 months improves feeding and social skills (Tauber et al., 2017).

### CNTNAP2

Missense heterozygous mutations in the contactin-associated protein-like 2 (*CNTNAP2*) are implicated in cortical dysplasia-focal epilepsy (CFDE) syndrome and are associated with epilepsy, seizures, attention-deficit hyperactivity disorder (ADHD) and ASD (Strauss et al., 2006; Elia et al., 2010; Mefford et al., 2010; Rodenas-Cuadrado et al., 2014). The *CNTNAP2* gene encodes for the CASPR2 protein, which is a member of the neurexin superfamily. Presynaptic neurexins interact with members of the neuroligin family at the post synapse, and members of both protein families have been associated with ASD (Betancur et al., 2009). Similar to human subjects, mice with a *Cntnap2* mutation also have epileptic seizures and show deficits in ASD-associated behaviors (Brunner et al., 2015; Peñagarikano et al., 2015). Notably, *Cntnap2*-KO mice exhibit a significant and specific reduction in the number of OXT expressing cells in the PVN as well as in OXT concentrations in brain extracts. Single intraperitoneal or intranasal application of OT in these mice is sufficient to transiently rescue their social behavior deficits. Interestingly, early postnatal sub-chronic intranasal application of OXT alleviates their social behavior deficits and restores PVN-OXT neurons and brain OXT concentrations to wild-type levels (Peñagarikano et al., 2015). Together, these findings suggest that the OXT system may be affected in individuals with *CNTNAP2* mutations and that those individuals may particularly benefit from an early-life treatment with OXT.

### Shank3

We have recently produced and validated the *Shank3*-deficient rat model, a novel transgenic rat model for ASD and intellectual disability that harbors a mutation in the *Shank3* gene (Harony-Nicolas et al., 2017). Shank3 is a scaffolding protein at the postsynaptic density (PSD), which functions as a platform upon which other additional PSD proteins accrete (Grabrucker et al., 2011). In humans, deletions or mutations in the *SHANK3* gene lead to Phelan-McDermid Syndrome (PMS) with approximately 80% meeting criteria for ASD (Soorya et al., 2013). Mouse models with *Shank3* gene mutations display ASD-related behavioral phenotypes, altered synaptic transmission and changes in neural plasticity and synaptic morphology (Harony-Nicolas et al., 2015). In rats, we found that *Shank3* mutations lead to deficits in attention, long- but not short-term SRM (Harony-Nicolas et al., 2017) and developmental social communication (Berg et al., 2018). We also found that these deficits are accompanied by impaired synaptic plasticity. Specifically, we showed that maintenance of LTP in the hippocampus (*in vitro*) and the hippocampal-PFC pathway (*in vivo*) is impaired in the *Shank3*-deficient rat. Finally, we demonstrated that acute ICV administration of OXT in these rats reversed both the behavioral and the *in vitro* and *in vivo* synaptic plasticity deficits (Harony-Nicolas et al., 2017). These findings were the first to report that OXT can reverse not only behavioral but also synaptic plasticity deficits in a genetic model for a neurodevelopmental disorder, suggesting that the reversal effect of OXT on synaptic plasticity, specifically LTP, may underlie its ameliorative effect on behavior. Further studies are needed to determine the effect of *SHANK3* mutations on the OXT system in order to understand if perturbation in this system could explain some of the observed behavioral phenotypes and plasticity-related changes.

## Concluding Remarks

Here, we provided an overview of the modulatory effects of OXT on synaptic activity that underlie diverse behaviors. We also reviewed findings from genetic rodent models of neurodevelopmental disorders that demonstrate alterations in the OXT system. Despite the major interest in the therapeutic potential of OXT to treat social behavior deficits, there is still a considerable gap in the knowledge about the plausible implication of the OXT system in the pathogenesis of neurodevelopmental disorders. To fill this gap, there is a need for future studies to investigate not only the effect of mutations associated with these disorders on the maturation of the OXT system during early development stages but also the integrity and functionality of this system during later postnatal windows and under different behavioral contexts. Many of the mutations associated with neurodevelopmental disorders reside in genes encoding for neural or synaptic proteins (Xu et al., 2012; De Rubeis et al., 2014; Sanders et al., 2015), suggesting that they may impact: (1) the development and maturation of OXT neurons; (2) the OXT projections; (3) the intrinsic properties of OXT neurons; and (4) the trafficking and release of OXT. An alteration in any of these elements could potentially lead to imbalanced OXT levels in the brain and/or a lack or diminished response by the OXT system to stimuli (e.g., stress or social stimuli). This consequence could affect synaptic activity and plasticity in target brain regions modulated by OXT and ultimately impair the behavioral response. The CD38^−/−^ mouse model provides an example for how behavioral phenotypes can be attributed to deficits in OXT release. This model harbors a mutation in the CD38 gene that encodes for a transmembrane glycoprotein involved in OXT release. CD38^−/−^ mice have reduced OXT plasma levels and increased number of large dense core vesicles (LDCVs), which package the OXT neuropeptide. These alterations are accompanied with impaired maternal nurturing and SRM, which can be rescued with OXT administration (Jin et al., 2007).

Our findings in the *Shank3*-deficient rat, where both the behavioral and the synaptic plasticity deficits are rescued with OXT administration (Harony-Nicolas et al., 2017), suggest that OXT delivery and/or release at target brain regions involved in social behavior may be impaired. Notably, OXT release is known to be reliant on rapid and transient depolymerization of actin filaments (Tobin et al., 2012), and *Shank3*-deficient mice show dysregulation of actin filaments via upregulation of cofilin, a known actin depolymerizing agent (Duffney et al., 2015). Based on these findings it is possible that mutations in the *SHANK3* gene would impact the actin cytoskeleton in OXT neurons, the release of OXT, and consequently, synaptic plasticity and behaviors that are modulated by OXT. To address this theory, there is a need for future studies to assess the central and peripheral OXT levels in this model and OXT levels following behavioral (e.g., social or stress stimuli), drug-induced (e.g., CCK8) or chemogenetic (designer receptors exclusively activated by designer drug; DREADDS) activation of OXT-neurons. Moreover, given that OXT neurons project to several brain targets, it is important to elucidate whether the effect of OXT on modulating synaptic activity persists across all projection targets to influence a circuit and whether some of these target regions are more vulnerable to genetic insults than others.

Importantly, given the etiological and phenotypic heterogeneity in ASD and neurodevelopmental disorders, we do not expect impairment in the OXT system to explain the behavioral phenotypes of all individuals with the disorder. This highlights the need for future studies in additional genetic models of neurodevelopmental disorders to (1) identify mutations that pose a deleterious effect on the OXT system and therefore converge on a shared pathophysiology and (2) to define the mechanistic interplay between these mutations and the OXT system. Findings from these studies will inform targeted treatments in human individuals carrying these pathogenic mutations.

## Author Contributions

KTR, SW, VG and HH-N contributed to the writing of the mini review.

## Conflict of Interest Statement

The authors declare that the research was conducted in the absence of any commercial or financial relationships that could be construed as a potential conflict of interest.
